# Incidence and predictors of post‐surgery atrial fibrillation occurrence: A cohort study in 53,387 patients

**DOI:** 10.1002/joa3.13058

**Published:** 2024-05-19

**Authors:** Enrico Brunetta, Guido Del Monaco, Stefano Rodolfi, Donah Zachariah, Kostantinos Vlachos, Alessia Chiara Latini, Maria De Santis, Carlo Ceriotti, Paola Galimberti, Antonio Taormina, Vincenzo Battaglia, Giulio Falasconi, Diego Penela Maceda, Michael Efremidis, Konstantinos P. Letsas, Carlo Selmi, Giulio Giuseppe Stefanini, Gianluigi Condorelli, Antonio Frontera

**Affiliations:** ^1^ Unit of Rheumatology and Clinical Immunology, IRCCS Humanitas Research Hospital Rozzano Italy; ^2^ Arrhythmology Department, IRCCS Humanitas Research Hospital Rozzano Italy; ^3^ Department of Biomedical Sciences Humanitas University, Pieve Emanuele Milan Italy; ^4^ Cardio Center, Humanitas Clinical and Research Hospital IRCCS Milan Italy; ^5^ Department of Cardiac Electrophysiology Royal Papworth Hospital Cambridge UK; ^6^ Onassis Centre Athens Greece

**Keywords:** atrial fibrillation, cardioversion, post‐operative, surgery

## Abstract

**Introduction:**

Atrial fibrillation (AF) represents the most common arrhythmia in the postoperative setting. We aimed to investigate the incidence of postoperative AF (POAF) and determine its predictors, with a specific focus on inflammation markers.

**Methods:**

We performed a retrospective single tertiary center cohort study including consecutive adult patients who underwent a major surgical procedure between January 2016 and January 2020. Patients were divided into four subgroups according to the type of surgery.

**Results:**

Among 53,387 included patients (79.4% male, age 64.5 ± 9.5 years), POAF occurred in 570 (1.1%) with a mean latency after surgery of 3.4 ± 2.6 days. Ninety patients died (0.17%) after a mean of 13.7 ± 8.4 days. The 28‐day arrhythmia‐free survival was lower in patients undergoing lung and cardiovascular surgery (*p* < .001). Patients who developed POAF had higher levels of C‐reactive protein (CRP) (0.70 ± 0.03 vs. 0.40 ± 0.01 log10 mg/dl; *p* < .001). In the multivariable Cox regression analysis, adjusting for confounding factors, CRP was an independent predictor of POAF [HR per 1 mg/dL increase in log‐scale = 1.81 (95% CI 1.18–2.79); *p* = .007]. Moreover, independent predictors of POAF were also age (HR/1 year increase = 1.06 (95% CI 1.04–1.08); I < .001), lung and cardiovascular surgery (HR 23.62; (95% CI 5.65–98.73); *p* < .001), and abdominal and esophageal surgery (HR 6.26; 95% CI 1.48–26.49; *p* = .013).

**Conclusions:**

Lung and cardiovascular surgery had the highest risk of POAF in the presented cohort. CRP was an independent predictor of POAF and postsurgery inflammation may represent a major driver in the pathophysiology of the arrhythmia.

## Graphical abstract

In the present study, POAF represents a frequent complication following several types of surgery (stratified into four groups) with a higher incidence after cardiac and thoracic surgery. In our casuistic inflammation (represented by CRP levels) is an independent predictor of POAF occurrence.

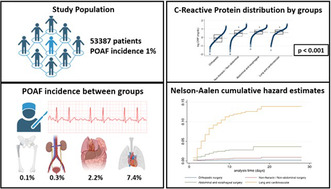

## 1 INTRODUCTION

Atrial fibrillation (AF) is the most common sustained cardiac arrhythmia in the adult population and is known to be associated with increased morbidity and mortality. The estimated prevalence of AF in adults ranges between 2% and 4% worldwide and is expected to rise in the next years due to the aging population and the increase in diagnosis rate.^1^, ^2^ Postoperative AF (POAF) is defined by the Society of Thoracic Surgeons (STS) as “the new onset of atrial fibrillation or atrial flutter after surgery requiring treatment” and has a peak of incidence between days 0 and 5 after surgery.^3^ Despite being classically considered a self‐limiting phenomenon, recent evidence suggests that POAF is associated with postoperative morbidity, mortality, length of stay, ICU admission, thromboembolic events, stroke, and hospitalizations due to heart failure.^4^ Cardiac surgery carries the greatest risk of POAF, reporting an incidence from 20% to 40%, with a risk substantially higher for valve surgery compared to coronary artery bypass graft (CABG).^5^, ^6^ POAF may also occur after thoracic surgery, especially after esophagectomy and lung surgery.^7^, ^8^ By contrast, the incidence after noncardiac nonthoracic surgery is significantly lower (about 2.2%), and, amongst all surgical categories, large abdominal surgery carries the highest rates of POAF.^9^ However, the causes of the differences between the risks of POAF according to the type of surgery remain unclear. It has been hypothesized that inflammation could play a role in the pathophysiology of POAF, likely by favoring ectopic firing and subsequent promoting maintenance of arrhythmia and disorganized atrial activity.^10^

The aim of this cohort study is to investigate the incidence of postoperative AF (POAF) and determine its predictors, with a specific focus on inflammation markers.

## 2 METHODS

### 2.1 Patient sample

Adult patients (≥18 years) admitted to IRCCS Humanitas Research Hospital (Rozzano, Milan, Italy) between January 2016 and January 2020 for any surgery procedure were retrospectively included in our first cohort of 53,387 patients. AF was defined according to the latest guidelines as a 12‐lead ECG recording or a single‐lead ECG tracing of ≥30 s showing heart rhythm with no discernible repeating *P* waves and irregular RR interval.^2^ POAF was defined as AF diagnosed within 28 days after surgery.^3^ Based on the type of surgery performed, in order to evaluate the potential impact of different involvement of heart and surrounding structures, patients were classified into four groups: (I) orthopedic surgery, (II) nonthoracic nonabdominal surgery, (III) abdominal and esophageal surgery, (IV) lung and cardiovascular surgery. Data on medication were collected from each patient chart and included class Ia, Ic, or III antiarrhythmic drugs according to the Vaughan–Williams classification. Laboratory tests performed within 48 h postsurgery were included in the analysis. In particular, to ensure uniformity in the selected population and to allow a correlation between inflammation and incidence of POAF, C‐reactive protein (CRP) was consistently measured within 48 h of the surgical intervention. Patients with CRP measurements only after 48 h were excluded, as were those with documented infectious complications during the postintervention period (i.e. positive blood cultures or urine cultures). Data on vital parameters such as body temperature, blood pressure, heart rate, peripheral saturation, and respiratory rate were obtained from in‐patient observation charts in all patients. All the data described above and a comprehensive current and past medical history were extracted from electronic medical records and checked by a team of three expert physicians. The study complied with the Declaration of Helsinki and was approved by the Institutional Ethics Committee.

### 2.2 Statistical methods

A standardized data collection form was used to record the demographic, clinical, and laboratory. Descriptive statistics included means with standard deviations (SD) and medians with interquartile ranges (IQR) for continuous variables as appropriate, and frequency analyses (percentages) for categorical variables. Wilcoxon rank‐sum tests (for continuous variables) and Fisher's exact tests (for categorical variables) were applied. The linearity of continuous variables was checked by comparing models with the linear term to the model with restricted cubic splines. Because of nonlinearity, CRP was used in the logarithmic scale for regression analysis, to make the distribution more symmetric and to reduce the effect of outliers. The choice to use the logarithmic transformation of the CRP variable was made to improve the validity and predictive capability of the Cox regression model. To identify the association between CRP levels and the outcome in patients, we used time‐to‐event methods for censored observations. In the context of our study, we specifically chose orthopedic surgery to serve as the reference group for comparative analysis. This decision was based on the understanding that orthopedic surgical procedures typically involve less manipulation of both parenchymal tissues and serous membranes, making it an ideal baseline against which to measure and contrast the impacts of other surgical interventions under investigation. The study endpoint was the onset of new AF within 28 days of surgery. Time‐to‐event was defined as the time from the day of surgery until the date of the event or censoring. Patients discharged before 28 days with no documented AF onset could not be considered event‐free through day 28. Kaplan–Meier estimates were used to draw the cumulative incidence curves by surgery groups, and then the curves were compared with a log‐rank test. In addition to the Kaplan–Meier method, the Nelson‐Aalen method is used to estimate the cumulative incidence function of POAF in competition with survival. A multivariable Cox proportional hazards (PH) model of prognostic factors was used.

The analyses were based on nonmissing data (missing data not imputed). Confounders were selected according to a review of literature, statistical relevance, and consensus opinion by an expert group of physicians and methodologists. Gender was excluded because of its irrelevance in modifying the HR in the analysis (HR modification was <10% when gender was included in a CRP univariable Cox model). The multivariate Cox with adjustment of the odds ratio for the type of intervention, as well as for age, and hospitalization duration was used to establish a correlation between CRP levels and the incidence of POAF regardless of the type of surgical intervention. After fitting the model, the PH assumption was examined on the basis of Schoenfeld residuals. The hazard ratios (HR) were presented with their 95% confidence intervals (CI) and the respective *p*‐values. A ratio higher than 1.0 implies a higher probability of new onset of AF, compared to the reference group. The statistical significance level was set at a two‐sided p‐value of less than 0.05, indicating a threshold for significance. All data were formatted using R Studio (version 3.6.2, R Foundation for Statistical Computing, Vienna, Austria.), and statistical analyses were performed using Stata 15.1. (StataCorp 4905 Lakeway Dr College Station, TX 77845 USA).

## 3 RESULTS

**FIGURE 1:**
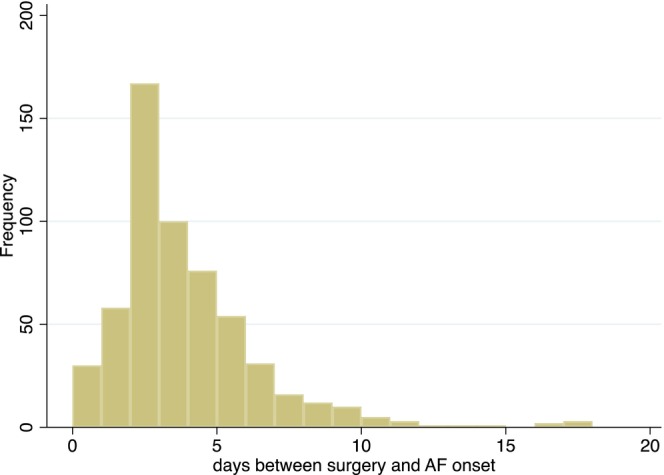
Distribution of postoperative atrial fibrillation (POAF) incidence over postoperative days.

**FIGURE 2:**
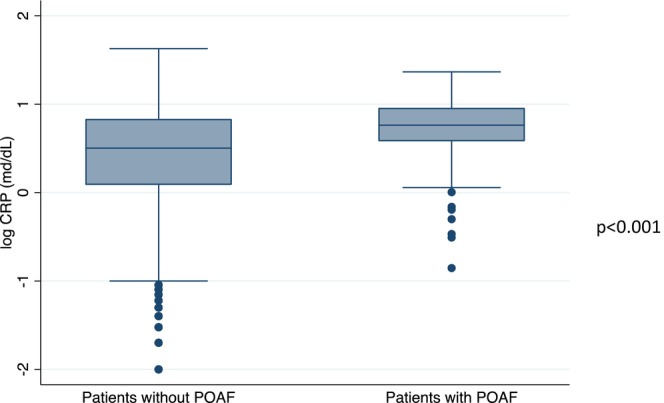
(A) Analysis of plasma C‐reactive protein (CRP) levels in patients stratified by postoperative atrial fibrillation (POAF) occurrence. (B) Plasma CRP levels stratified by surgery type, *p < .001. (C) Distribution of plasma CRP levels in patients with POAF stratified by surgery type.

The demographic, clinical, and laboratory characteristics of the patients are shown in Table 1. POAF occurred in 570 patients (1.1%), the mean duration of hospital stay was 5.5 ± 4.1 days (median 4 days, IQR 3–7 days) while the mean interval between surgery and the new onset of POAF was 3.4 ± 2.6 days (median 3 days, IQR 2–4 days). Among patients who developed AF, 532 patients (93.3%) had an onset in the first week after surgery, 33 patients (5,8%) in the second week, and 5 patients (0.88%) in the third week (Figure 1). Ninety patients (0.17%, 4 of whom had developed POAF) died after an average of 11.3 ± 8.0 days (median 10 days, IQR 5–19 days) after surgery, A total of 1973 patients had a prior history of atrial arrhythmias and 349 (18%) of these developed POAF during their hospital stay. The distribution of POAF events by surgery groups is described in Table 2. Serum CRP levels within 48 h from surgery were available in 9426 of 53,387 patients of our cohort (18%) and 140 of 9426 (1.5%) developed POAF; these patients had higher CRP compared to the remaining population (mean 0.70 ± 0.03 mg/dL vs. 0.40 ± 0.01 mg/dL; logarithmic scale; *p* < .001) (Figure 2A). When we compared CRP levels across surgery groups, a higher level of CRP was observed in patients who underwent lung and cardiovascular or abdominal and esophageal procedures (Figure 2B). However, when CRP levels were stratified by the type of surgery and in the POAF subgroup, there was no evidence of a significant variation between the groups (Figure 2C). We applied the Wilcoxon rank‐sum test to individual pairs within each group, ensuring that each test included a group of orthopedic surgery as a constant. In Figure 2B, the difference between groups remained significant (*p* < .001), while in Figure 2C, the difference between groups was not statistically significant.

**TABLE 1:** Demographic, clinical, and laboratory characteristics of patients (*n* = 53,387).

Variables	Orthopedic surgery	Nonthoracic nonabdominal surgery	Abdominal and esophageal surgery	Lung and cardiovascular surgery
Demographic				
Number of patients	17′205 (33.3%)	28,026 (52.5%)	2636 (4.9%)	5520 (10.3%)
Age (years)	59.8 ± 17.6	57.2 ± 15.6	62.2 ± 13.3	64.4 ± 13.4
Length of stay (days)	5.29 ± 3	4.9 ± 4	8.7 ± 5.8	7.8 ± 5.1
Males	8121 (47.2%)	13′424 (47.9%)	1471 (55.8%)	3063 (55.5%)
Females	9084 (52.8%)	14,602 (52.1%)	1165 (44.2%)	2457 (44.5%)
Clinical				
Oral antiarrhythmic usage	495 (2.8%)	587 (2.1%)	102 (3.9%)	789 (14.3%)
Days after surgery of AF onset (median, IQR)	1 (0–2)	4 (2–6)	3 (2–5)	3 (2–5)
Biochemical				
Leucocytes (cells/mm^3^)	11,269 ± 3409	11,459 ± 3709	10,093 ± 3910	11,019 ± 3929
Hemoglobin (g/dL)	11.73 ± 1.53	12.14 ± 1.67	11.36 ± 1.82	11.81 ± 1.59
Platelets (cell/mm3)	210 ± 58	223 ± 70.4	193 ± 71	191 ± 79
Creatinine (mg/dL)	0.81 ± 0.33	0.84 ± 0.56	0.84 ± 0 0.44	0.95 ± 0.52
Brain natriuretic peptide (pg/mL)	321.6 ± 264	251.9 ± 371	163.6 ± 125.6	368.8 ± 608.1
Lactate dehydrogenase (UI/L)	207.8 ± 69.7	267.8 ± 123	739 ± 755	451.6 ± 322.6

Abbreviations: AF, atrial fibrillation; Q1–Q3: (25–75th percentile).

**TABLE 2:** Distribution of the POAF events by surgery groups.

Group	Total patients	No POAF (%)	POAF (%)
Orthopedic surgery	17,205	17,182 (99,8)	23 (0.13)
Nonthoracic and nonabdominal surgery	28,026	27,946 (99,7)	80 (0.29)
Abdominal and esophageal surgery	2636	2579 (97.84)	57 (2.16)
Lung and cardiovascular surgery	5520	5110 (98.93)	410 (7.43)
	53,387	52,817	570

**FIGURE 3:**
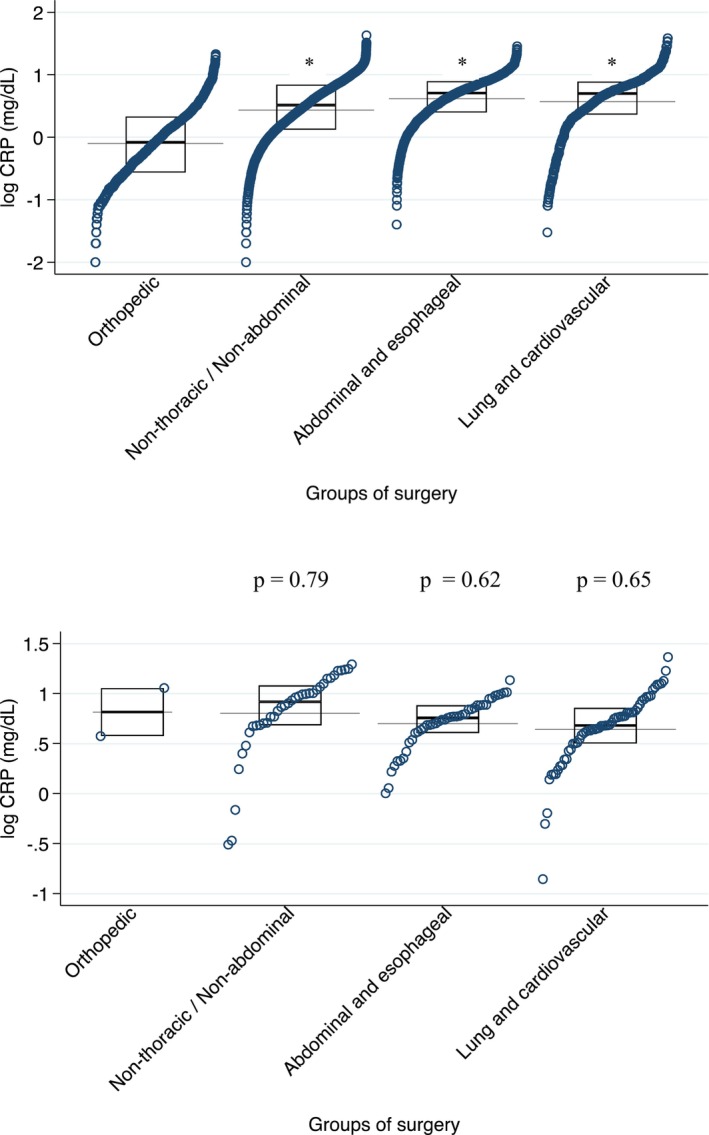
Cumulative hazard estimates in patients with postoperative atrial fibrillation (POAF) stratified by different types of surgery. Log‐rank *p*‐value <.001.

The Kaplan–Meier analysis showed an overall 28‐day event‐free survival of 0.998 (95% CI, 0.996–0.966) following orthopedic surgery, 0.99 (95% CI, 0.983–0.994) following nonthoracic nonabdominal surgery, 0.964 (95% CI, 0.947–0.975) after abdominal and esophageal surgery and 0.869 (95% CI, 0.847–0.888) in patients who underwent lung and cardiovascular surgery (log‐ranks test *p* < .001; Figure 3). CRP was associated with AF in patients who underwent surgery (HR per 1 mg/dL increase in log‐scale, 2.64; 95% CI, 1.74–4.0; *p* < .001) at the univariable Cox regression. After adjusting for age, gender, length of stay in hospital, and group of surgery, CRP was confirmed to be an independent predictor of POAF (adjusted HR [aHR] per 1 mg/dL increase in log‐scale, 1.81; 95% CI, 1.18–2.79; *p* = .007) (Table 3). Age was also an independent predictor of POAF (HR per 1 year increase, 1.06; 95% CI, 1.04–1.08; *p* < .001) as well as the type of surgery. In this last case, patients undergoing lung and cardiovascular surgery or abdominal and esophageal surgery were at the highest risk of developing POAF when compared to orthopedic surgery (HR 23.62; 95% CI, 5.65–98.73; *p* < .001 and HR 6.26; 95% CI, 1.48–26.49; *p* = .013, respectively). The PH assumption was not violated (*p* = .12). A sensitivity analysis of patients without any prior history of AF or of use of antiarrhythmic drugs confirmed the same results **(**Supplementary Table 1 **)**.

**TABLE 3:** Predictors of POAF at the multivariable analysis.

	Multivariable cox PH model		
Variable	Adjusted HR	(95% CI)	*p*‐value
Age (per 1 year increase)	1.06	(1.04–1.08)	<0.001
Gender (male vs. female)	1.25	(0.87–1.77)	0.22
C‐reactive protein log (per 1 mg/dL increase)	1.81	(1.18–2.18)	0.007
Hospitalization length (per 1 day increase)	4.81	(1.16–19.86)	0.03
Group of surgery (compared to orthopedic surgery)			
Nonthoracic and non‐abdominal surgery	2.37	(0.56–10.06)	0.24
Abdominal and esophageal surgery	6.26	(1.48–26.48)	0.013
Lung and cardiovascular surgery	23.62	(5.65–98.73)	<0.001

## 4 DISCUSSION

Increased CRP levels have been reported as a predictor of AF development in large prospective cohorts both in the postoperative setting and in the general population, and several case–control studies have reported higher levels of inflammatory markers (such as IL‐6, CRP, TNF and IL‐8).^11^, ^12^ Data from cellular and animal studies speculated that inflammation is not a mere bystander in POAF but contributes directly to its pathophysiology, by promoting structural remodeling, favoring ectopic firing, and generating re‐entry.^13^ Inflammation in POAF has been proposed to induce endothelial dysfunction, platelet activation, and a hypercoagulable state.^14^ Of note, pericardial fat has been identified as a source of proinflammatory cytokines.^15^, ^16^ Our data from a large cohort of patients undergoing different types of surgery confirmed the association between specific types of interventions and the incidence of POAF. However, as reported in the literature, the association with inflammation, represented by CRP levels, is independent with respect to the type of surgery.^17^

According to available studies, the incidence of POAF varies from 5% to 15% depending on the type of gastrointestinal intervention performed.^18^ The results of our study suggest drivers beyond a mere proinflammatory milieu; the observed differences in POAF rates cannot be fully explained but we hypothesize that greater manipulation of serous membranes or intraperitoneal viscera leads to a significant release of proinflammatory cytokines which may in turn, trigger and sustain atrial fibrillation, e.g. the occurrence of POAF after radical cystectomy has been reported as 5.3%,^19^ whereas with less invasive prostatectomy, it is only 3%.^20^ We hypothesize that this observation may be justified by inflammation induced by the manipulation of serous membranes; however, we acknowledge that our findings represent a correlation, not establishing causation. However, confirming causation between these events would require a different study design, and in our retrospective cohort study, we can only observe this association while contemplating a potential causative hypothesis. Recent studies have suggested a putative role for the activation of the NLRP3‐inflammasome involved in autoinflammatory diseases and in the pathogenesis of atrial fibrillation^21^ with an increased activation in atrial samples of animal models of AF. Moreover, its inhibition was associated with a reduction of rate of spontaneous atrial contractions and AF onset.^22^ NLRP3 inflammasome activity has been found to be greater in cardiomyocytes of patients with rheumatoid arthritis and a history AF compared to sinus rhythm controls. NLRP3 activation by gut microbiome has also been proposed in the pathogenesis of age‐related AF.^23^

Following the interest in inflammation and AF, several trials have been performed in an attempt to reduce the development/recurrence of POAF by acting on the inflammatory burst following surgery. Glucocorticoids have been studied in POAF and AF recurrence after catheter ablation, with contrasting results in randomized controlled trials.^24^, ^25^ A 2021 meta‐analysis evaluating 14 randomized double‐blind trials documented a beneficial effect of steroid therapy in reducing the rate of postoperative AF with no difference in intensive care unit and overall hospital stay.^26^ Colchicine acts on the degranulation of neutrophils and is currently used in numerous autoinflammatory conditions while being the only immunomodulatory drug studied in the setting of prevention of POAF after cardiac surgery. Several RCTs have evaluated the role of colchicine for prevention of postcardiothoracic surgery AF. The Colchicine for the Prevention of the Post cardiotomy Syndrome (COPPS) Atrial Fibrillation Sub study showed a reduced incidence of postoperative AF in patients treated with colchicine for 1 month after pericardiotomy.^27^ A 2017 meta‐analysis evaluating five randomized controlled trials showed that, compared to placebo or usual care, colchicine reduced POAF incidence by 30% and mean hospital stay by 1.2 days with a significant incidence in drug‐related adverse events but with no major adverse events.^28^ Despite the conflicting evidence, the current AHA/ACC/HRS Guidelines for the management of patients with AF give a class IIb recommendation (level of evidence: B) for the use of post‐operative colchicine for prevention of POAF after cardiac surgery.^1^ Finally, Canakinumab for the Prevention of Recurrences After Electrical Cardioversion in Patients With Persistent Atrial Fibrillation (CONVERT‐AF) evaluated IL‐1β blockade with canakinumab versus placebo in 24 patients with persistent AF and baseline CRP >1.25 mg/dL undergoing external electrical cardioversion (ECV). Despite a lower trend in the canakinumab arm, the primary endpoint of 6‐month AF recurrence was not reached. No significant adverse event (only 1 infection‐related hospitalization) was observed in the treatment group.^29^

Long‐term outcomes of patients with POAF remain largely unknown and a recent meta‐analysis of patients with POAF followed up for at least 12 months, showed that the development of POAF conferred a 4‐fold increased risk of stroke in the long term compared to those without POAF.^30^ Anticoagulation in POAF should be initiated based on CHA_2_DS_2_VASc scores and should be no different compared to routine AF management. Data about AF recurrence in this study was too heterogeneous to make any meaningful conclusions and although POAF is considered as a transient entity, for reasons highlighted above, further prospective long‐term studies are required to understand the risks of recurrence further.

### 4.1 Study limitations

The first limitation of this study is the retrospective nature of the presented single‐center study. Secondly, the definition of POAF was based only on a spot 12‐lead ECG, but the true incidence of AF is likely underestimated because continuous monitoring of cardiac rhythm was not available for most patients. Length of stay on average was 4–5 days, but as POAF is defined as AF occurring within 28 days of surgery, the shorter period of monitoring is likely to underestimate the true incidence of POAF. Thirdly, despite this study was conducted on the largest cohort analyzed in the current literature (53,387 patients), in the final analysis, CRP values were considered only for 9426 patients and, as explained in the methods, were obtained within 48 h of surgical intervention (mean 22.5 ± 5.71 h). While this criterion ensures uniformity in inflammation values related to surgical intervention, it has restricted the data usable for the study. Additionally, the length of hospitalization has affected this data, limiting the number of laboratory samples meeting our inclusion criteria, especially in surgical interventions with a short hospital stay. However, we believe that the number of patients analyzed in this subgroup remains substantial and valid for expressing a reliable correlation between CRP and the onset of POAF. Another limitation of our study is that the multivariate analysis wasn't adjusted for comorbidities or composite indexes. This limitation is primarily attributed to the retrospective nature of our study. Moreover, detailed echocardiographic data such as left ventricular ejection fraction, diastolic function, or left atrial size, which would have provided helpful information for our findings, were not examined.

## 5 CONCLUSIONS

POAF represents a frequent complication following several types of surgery, and poses a major burden for healthcare. Inflammation, herein represented by CRP levels, may be a major driver in POAF pathophysiology in cardiac and non‐cardiac surgery where mechanical manipulation of the serous membrane is necessary. This may explain the poor response to antiarrhythmic drugs and the often‐reported self‐limiting nature of the process.

## FUNDING INFORMATION

None.

## CONFLICT OF INTEREST STATEMENT

None.

## ETHICS STATEMENT

This study was performed in line with the principles of the Declaration of Helsinki. Approval was granted by the local Ethics Committee.

## PATIENT CONSENT STATEMENT

N/A.

## CLINICAL TRIAL REGISTRATION

N/A.

## Supporting information


**Supplementary Table 1.** Predictors of POAF at the multivariable analysis in patients without any prior history of AF or use of antiarrhythmic drugs.
