# Supplementary material for: Incidence and predictors of post‐surgery atrial fibrillation occurrence: A cohort study in 53,387 patients
Source: J Arrhythm. 2024 May 19;40(4):815–21. doi: 10.1002/joa3.13058 (PMC11317654; doi:10.1002/joa3.13058)
Supplement: Supplementary file 1 — Supplementary Table 1. Predictors of POAF at the multivariable analysis in patients without any prior history of AF or use of antiarrhythmic drugs. [file JOA3-40-815-s001.docx]

**Supplementary Table 1**. Predictors of POAF at the multivariable analysis in patients without any prior history of AF or of use of anti-arrhythmic drugs

|  | **Multivariable Cox PH Model** | | |
| --- | --- | --- | --- |
| **Variable** | **Adjusted HR** | **(95% CI)** | ***p*-value** |
| Age (per 1 year increase) | 1.06 | (1.04–1.08) | <0.001 |
| Gender (male vs. female) | 1.25 | (0.86–1.82) | 0.24 |
| C-reactive protein log (per 1 mg/dL increase) | 1.98 | (1.24–3.16) | 0.004 |
| Hospitalization length (per 1 day increase) | 4.51 | (1.09–18.69) | 0.038 |
| Group of surgery (compared to orthopedic surgery)  Non thoracic and non-abdominal surgery | 2.18 | (0.51–9.28) | 0.29 |
| Abdominal and esophageal surgery | 5.29 | (1.24–22.56) | 0.024 |
| Lung and cardiovascular surgery | 19.49 | (4.62–82.13) | <0.001 |

In the present study POAF represents a frequent complication following several types of surgery (stratified in four groups) with an higher incidence after cardiac and thoracic surgery. In our casuistic inflammation (represented by CRP levels) is an independent predictor of POAF occurrence.
